# Bioadhesive Alginate/Chitosan Hydrogels for Sustained Probiotic Delivery and Enhanced Antibacterial Treatment of Oral Ulcers

**DOI:** 10.3390/gels12070581

**Published:** 2026-07-01

**Authors:** Zhixiong Yu, Wenlin Qin, Xuanhe Fu, Kebin Xu, Xin Huang, Jinfeng He, Xin Li

**Affiliations:** 1 Shenyang Key Laboratory of Prevention and Treatment of Systemic Important Diseases Associated with Oral Diseases, Shenyang 110034, China; yuzhixiong@symc.edu.cn (Z.Y.); 19850566276@163.com (W.Q.); fuxuanhe@symc.edu.cn (X.F.); xukebin@symc.edu.cn (K.X.); huangxin951220@163.com (X.H.); 17379013103@163.com (J.H.); 2 School of Stomatology, Shenyang Medical College, Shenyang 110034, China; 3 Key Laboratory of Human Ethnic Specificity and Phenomics of Critical Illness in Liaoning Province, Shenyang 110034, China; 4 Liaoning Provincial Belt and Road Joint Laboratory for China-Pakistan Human Phenomics Research, Shenyang 110034, China; 5 Key Laboratory of Phenomics in Shenyang, Shenyang 110034, China

**Keywords:** probiotic, sodium alginate, chitosan, hydrogel, recurrent aphthous ulcer

## Abstract

Recurrent aphthous ulcers (RAU) are highly prevalent oral mucosal lesions, while current therapies are limited by short residence time and poor efficacy in the dynamic oral environment. Probiotics such as *Lactobacillus reuteri* (*L. reuteri*) exhibit antimicrobial, immunomodulatory, and tissue-repair functions; however, their direct application is restricted by low stability and retention in the oral cavity. In this study, *L. reuteri*-loaded sodium alginate/chitosan composite hydrogels were developed via ionic crosslinking as a bioadhesive platform for local delivery. The hydrogels exhibited well-defined porous structures, favorable viscoelastic properties, and tunable swelling and degradation behaviors under simulated physiological conditions. Importantly, the system enabled sustained probiotic release and maintained high viability during 30 days of storage. In vitro antibacterial assays demonstrated that the hydrogels effectively inhibited the growth of *Staphylococcus aureus* and *Candida albicans*. Among the formulations, the hydrogel with 10 mg·mL^−1^ chitosan achieved an optimal balance between structural stability and mass transfer, resulting in enhanced release performance and antibacterial efficacy. Overall, this study presents a microecology-oriented hydrogel system for efficient probiotic delivery in oral environments, offering a promising bioadhesive probiotic delivery platform with potential applications in RAU management and advancing the development of bioactive, mucosa-adaptive therapeutic platforms.

## 1. Introduction

Recurrent aphthous ulcers (RAU) are one of the most common oral mucosal disorders, affecting approximately 20–25% of the population and significantly impairing quality of life due to recurrent pain and inflammation [1,2,3]. Although topical anti-inflammatory agents and corticosteroids are widely used, their therapeutic efficacy is often limited by rapid clearance in the dynamic oral environment, leading to short residence time and unsatisfactory clinical outcomes [4,5,6]. Moreover, prolonged drug use may cause adverse effects such as mucosal atrophy and microbial imbalance [7,8]. Therefore, developing localized delivery systems with prolonged retention and multifunctional therapeutic effects is highly desirable.

Probiotics are living microorganisms generally recognized as safe, capable of antagonizing pathogens and conferring health benefits to the host [9]. Among them, *Lactobacillus reuteri* (*L. reuteri*) is one of the most extensively studied strains, known for promoting gastrointestinal health, modulating immune responses, and exhibiting antifungal activity [10,11]. In oral disease management, probiotics exert therapeutic effects through multiple mechanisms, including inhibition of pathogenic microorganisms, secretion of antimicrobial substances, reduction in local pH to suppress inflammation, and modulation of host immune responses [12,13]. However, the complex oral environment, including salivary clearance, enzymatic degradation, and oxidative stress, significantly limits the survival and colonization of free probiotics [14]. Therefore, an effective encapsulation strategy is required to preserve probiotic viability, enable prolonged oral retention, and facilitate sustained co-delivery [15].

Bioadhesive hydrogels, with their hydrophilic three-dimensional network structure, excellent biocompatibility, and strong adhesion to moist mucosal tissues, have been widely explored for oral drug delivery [16,17]. These systems can conform to irregular ulcer surfaces, form protective barriers, and facilitate sustained release of therapeutic agents, thereby overcoming the limitations of conventional formulations [5]. Sodium alginate (SA), an anionic natural polysaccharide, exhibits excellent biosafety and mild ionic crosslinking capability, making it suitable for encapsulating sensitive bioactive substances such as probiotics [18]. In contrast, chitosan (CS), a cationic polysaccharide, possesses inherent mucoadhesive, antimicrobial, and wound-healing properties [19]. The electrostatic interaction between SA and CS leads to the formation of polyelectrolyte complexes, generating a denser interpenetrating network compared to single-component hydrogels, with improved mechanical strength and biological functionality [20,21]. Recent studies have further demonstrated that chitosan-based crosslinked polymeric networks possess tunable porosity and functional properties, which are closely related to their network structure and intermolecular interactions [22]. Importantly, the physicochemical properties of SA/CS hydrogels are highly dependent on CS content, which governs network architecture, pore structure, swelling behavior, and mass transport characteristics. This compositional tunability further enables controlled drug release and pH-responsive behavior in the weakly acidic microenvironment of oral ulcers [23,24].

Despite increasing interest in SA/CS composite hydrogels, their application as probiotic delivery systems for oral mucosal diseases remains limited, particularly regarding the relationship between composition, microstructure, and functional performance. Previous studies have reported the use of SA/CS-based hydrogels for probiotic encapsulation and delivery [25,26]. However, these studies have mainly focused on encapsulation efficiency and probiotic viability, while systematic investigations of how compositional variation influences microstructure and multi-functional performance remain insufficient [27,28]. In this study, five *Lactobacillus reuteri*-sodium alginate/chitosan (*L. reuteri*-SA/CS1-5) hydrogels with varying chitosan concentrations were fabricated via ionic crosslinking. The effects of CS content on microstructure, intermolecular interactions, viscoelastic properties, swelling behavior, and degradation were systematically investigated. Furthermore, probiotic release, storage stability, and antibacterial activity against *Staphylococcus aureus* and *Candida albicans* were evaluated. This work aims to elucidate the structure, property, and function relationship of SA/CS hydrogels and provide a theoretical basis for their application as probiotic delivery systems for oral mucosal delivery and future ulcer-healing applications.

## 2. Results and Discussion

### 2.1. Preparation of Hydrogel

**Figure 1:**
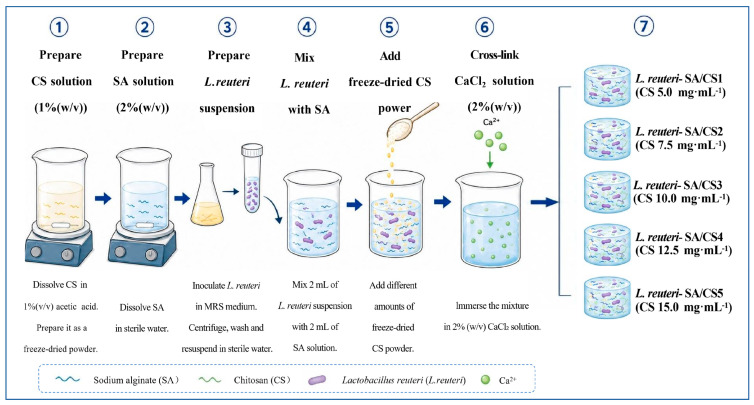
Schematic illustration of the preparation of *L. reuteri*-SA/CS1-5 hydrogels.

To facilitate understanding of the experimental design before data interpretation, a schematic illustration of hydrogel preparation and sample designation is presented in Figure 1. Five *L. reuteri*-loaded sodium alginate/chitosan hydrogels were fabricated with identical sodium alginate and probiotic contents, while chitosan concentration was varied as the sole formulation parameter. The resulting formulations were designated as *L. reuteri*-SA/CS1-5 hydrogels.

**Figure 2:**
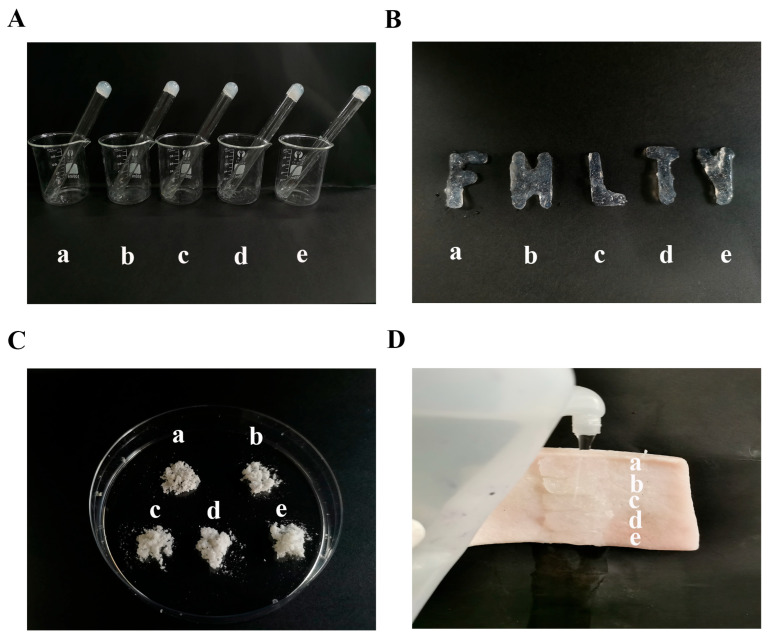
Morphology and adhesive properties of *L. reuteri*-SA/CS1-5 hydrogels. (**A**) Inverted tube test showing gel formation; (**B**) Hydrogels molded into different shapes; (**C**) Lyophilized hydrogel samples; (**D**) Adhesion behavior on porcine skin. Samples a–e correspond to *L. reuteri*-SA/CS1-5 hydrogels, respectively.

The *L. reuteri*-SA/CS1-5 hydrogels with different chitosan concentrations were successfully prepared by ionic crosslinking. The inverted tube test confirmed rapid gelation and good structural integrity, as no flow was observed upon inversion (Figure 2A). The hydrogels exhibited favorable plasticity, allowing molding into defined shapes (Figure 2B). After lyophilization, dried hydrogel matrices were obtained (Figure 2C). As shown in Figure 2D, the *L. reuteri*-SA/CS1-5 hydrogels adhered to the surface of porcine skin and remained attached after water rinsing, demonstrating qualitative wet-tissue adhesive capability. However, quantitative measurements of mucoadhesive strength were not performed in the present study. This property is mainly attributed to the presence of chitosan, whose cationic amino groups can interact with negatively charged mucosal surfaces, enhancing interfacial adhesion [17]. Combined with their structural stability, these characteristics suggest that SA/CS hydrogels are capable of maintaining prolonged residence at the application site in the dynamic oral environment, thereby facilitating sustained drug release and improved therapeutic efficacy.

### 2.2. Characterization of Hydrogel

**Figure 3:**
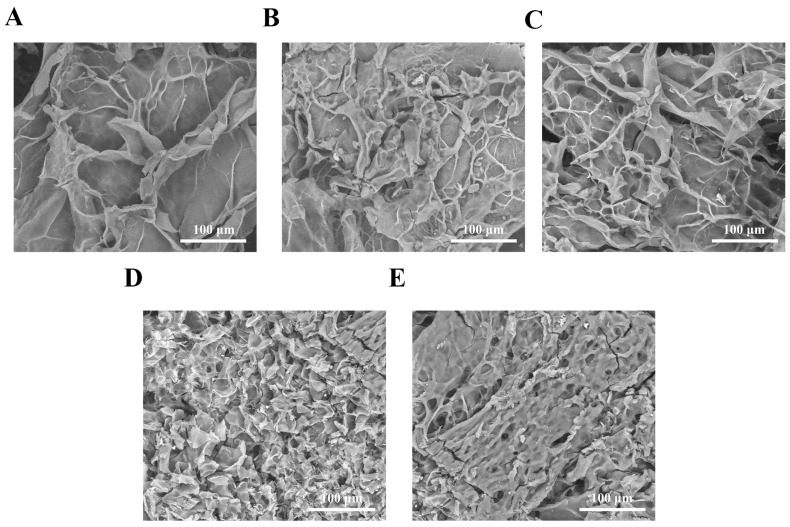
Scanning electron microscope (SEM) images of *L. reuteri*-SA/CS1-5 hydrogels with different chitosan concentrations. (**A**) *L. reuteri*-SA/CS1; (**B**) *L. reuteri*-SA/CS2; (**C**) *L. reuteri*-SA/CS3; (**D**) *L. reuteri*-SA/CS4; (**E**) *L. reuteri*-SA/CS5.

The micromorphology of *L. reuteri*-SA/CS1-5 hydrogels was characterized by scanning electron microscope (SEM) (Figure 3A–E). All formulations exhibited typical three-dimensional porous network structures. Increasing chitosan concentration markedly affected pore size. Quantitative measurements of representative pores from SEM micrographs showed that the average pore diameters of the *L. reuteri*-SA/CS1-5 hydrogels were 144.6 ± 15.9 μm, 127.9 ± 14.0 μm, 100.7 ± 12.4 μm, 79.1 ± 10.3 μm, and 58.9 ± 7.7 μm, respectively (Figure S1). *L. reuteri*-SA/CS1-3 hydrogels (5.0–10.0 mg·mL^−1^; Figure 3A–C) showed relatively large pores, whereas the *L. reuteri*-SA/CS4 and *L. reuteri*-SA/CS5 hydrogels (12.5 and 15.0 mg·mL^−1^; Figure 3D,E) exhibited reduced pore size, indicating network densification at elevated CS levels [29]. This phenomenon can be attributed to the enhanced electrostatic interactions between the protonated amino groups of chitosan and the carboxyl groups of alginate at higher CS concentrations, which increase the crosslinking density and restrict pore expansion while simultaneously reducing polymer chain mobility and accelerating polyelectrolyte complexation [30]. In addition, the rapid network formation limits phase separation and suppresses ice crystal growth during freeze-drying, resulting in smaller and less interconnected pores [31]. Similar trends have been reported in previous studies on alginate/chitosan composite hydrogels, where excessive chitosan content resulted in compact network structures with limited porosity [32]. Quantitative pore size distribution analysis further showed that the coefficient of variation (CV) values ranged from 10.9% to 13.1% among the different formulations, indicating that increasing CS concentration reduced the average pore diameter but had little effect on the relative dispersion of pore diameters (Figure S1).

**Figure 4:**
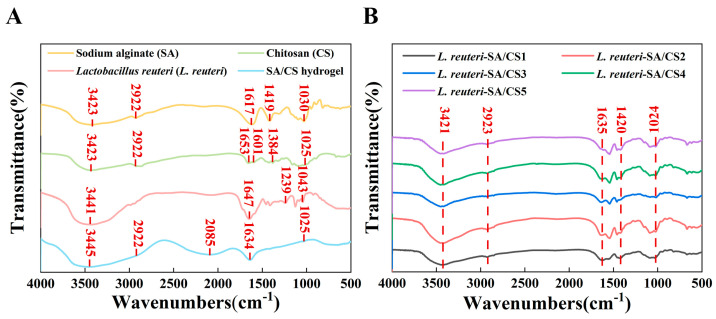
Fourier transform infrared (FTIR) spectra of the individual components and probiotic-loaded SA/CS hydrogels. (**A**) Sodium alginate (SA), chitosan (CS), *Lactobacillus reuteri* (*L. reuteri*), and the SA/CS hydrogel. (**B**) *L. reuteri*-SA/CS1-5 hydrogels.

Figure 4A shows the Fourier transform infrared (FTIR) spectroscopy of SA, CS, *L. reuteri*, and the SA/CS hydrogel. The FTIR spectra of SA exhibited characteristic absorption bands at 3423 cm^−1^ (O–H stretching), 1617 cm^−1^ and 1419 cm^−1^ corresponding to the asymmetric and symmetric stretching vibrations of carboxylate groups (COO^−^), respectively. CS showed characteristic peaks at 1653 cm^−1^ (amide I) and 1601 cm^−1^ (NH_2_ bending vibration), confirming its typical polysaccharide structure. In addition, bands at approximately 1024 cm^−1^ and 2923 cm^−1^ were observed in both SA and CS spectra, corresponding to the C–O–C stretching vibration of the polysaccharide backbone and the asymmetric stretching vibration of C–H bonds in –CH_2_– groups, respectively. The retention of these bands in the SA/CS hydrogel spectrum indicates preservation of the polysaccharide structure after complexation. After complexation, the band near 1647 cm^−1^, which may arise from overlapping contributions of COO^−^ asymmetric stretching and amide I vibrations, shifted relative to the corresponding bands of the individual components and exhibited changes in intensity. These spectral changes suggest electrostatic interactions between the carboxyl groups of SA and the protonated amino groups of CS, supporting the formation of a polyelectrolyte complex network [33,34].

As shown in Figure 4B, *L. reuteri*-SA/CS1-5 hydrogels exhibited similar FTIR profiles regardless of chitosan concentration. Compared with the SA/CS hydrogel, the FTIR spectra of *L. reuteri*-SA/CS1-5 hydrogels showed no obvious new absorption bands, while the major characteristic peaks of the hydrogel matrix were retained. This suggests that the incorporation of *L. reuteri* did not alter the chemical structure of the SA/CS network. Therefore, the FTIR results suggest that the incorporation of *L. reuteri* did not significantly alter the chemical structure of the SA/CS hydrogel network, while maintaining the integrity of the hydrogel structure [35]. Additional characterization techniques would be required to determine the spatial distribution of *L. reuteri* within the hydrogel system.

### 2.3. Rheological Property

**Figure 5:**
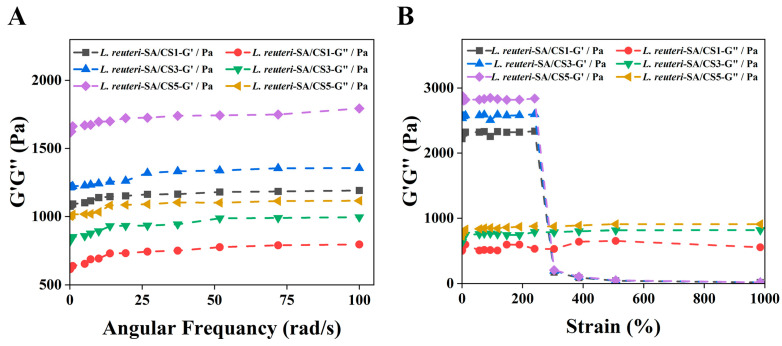
Rheological properties of *L. reuteri*-SA/CS1-5 hydrogels. (**A**) Storage modulus (G′) and loss modulus (G″) as functions of angular frequency; (**B**) Storage modulus (G′) and loss modulus (G″) as functions of strain.

The rheological properties of *L. reuteri*-SA/CS1, *L. reuteri*-SA/CS3, and *L. reuteri*-SA/CS5 hydrogels were evaluated at 37 °C using a rotational rheometer to assess their mechanical performance under physiological conditions. As shown in Figure 5A, frequency sweep analysis revealed that the storage modulus (G′) consistently exceeded the loss modulus (G″) over the angular frequency range of 0.1–100 rad·s^−1^, with both moduli exhibiting weak frequency dependence. This behavior indicates a predominantly elastic and solid-like viscoelastic nature, characteristic of a well-developed and dynamically stable three-dimensional network. Such behavior may be attributed to the formation of physical crosslinking points via electrostatic interactions between –NH_3_^+^ (CS) and –COO^−^ (SA), along with hydrogen bonding and polymer chain entanglement [36]. These cooperative interactions enable the hydrogels to maintain structural integrity and resist deformation under dynamic conditions.

As shown in Figure 5B, G′ remained relatively constant within the low-strain region and began to decrease noticeably beyond the critical strain range (approximately 200–300%, depending on formulation). This suggests that the hydrogel network can preserve its structure under moderate mechanical stress. When the strain exceeded the critical strain range, G′ intersected with G″ and subsequently decreased sharply, indicating the onset of network disruption and a gel–sol transition. This transition is associated with the progressive dissociation of physical crosslinks and disentanglement of polymer chains under high shear stress.

Notably, all formulations showed a predominantly elastic response, although the absolute modulus values and critical strain differed among samples. Slight variations in modulus values were observed, which may be attributed to differences in local network compactness and chain mobility induced by varying CS concentrations [37]. Overall, the hydrogels exhibit robust viscoelastic properties, including dominant elastic behavior, high structural stability, and resistance to mechanical deformation. These characteristics are essential for maintaining gel integrity under dynamic oral conditions, thereby supporting prolonged retention and sustained probiotic delivery at the target site.

### 2.4. Swelling Behavior

**Figure 6:**
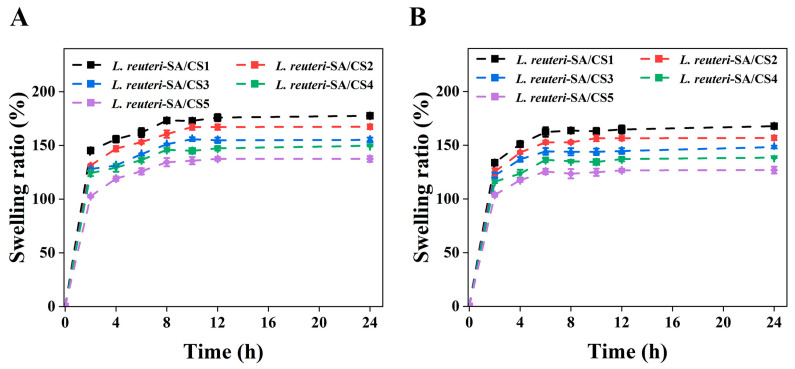
Swelling behavior of *L. reuteri*-SA/CS1-5 hydrogels. (**A**) Swelling in phosphate-buffered saline (PBS) buffer; (**B**) Swelling in artificial saliva.

The swelling behavior of *L. reuteri*-SA/CS1-5 hydrogels in Phosphate-buffered saline (PBS, pH 7.2) and artificial saliva (pH 6.8) is presented in Figure 6A,B. All formulations maintained structural integrity in both media and reached swelling equilibrium at approximately 8 h in PBS buffer and 6 h in artificial saliva, indicating the formation of stable crosslinked networks. The faster equilibration in artificial saliva is likely related to its higher ionic strength and the presence of multivalent ions, which may influence ion exchange interactions and promote additional ionic crosslinking with alginate, thereby affecting the hydration behavior and network stability of the hydrogels [38]. At equilibrium, the swelling ratios ranged from 137.6 to 177.6% in PBS buffer and 126.9–167.4% in artificial saliva. Overall, only modest differences were observed between the two media, with PBS generally showing slightly higher swelling ratios at the same time point. This difference is primarily attributed to the ionic composition of the swelling media. Specifically, divalent cations (e.g., Ca^2+^ and Mg^2+^) in artificial saliva can form additional ionic bridges with alginate chains, leading to a more compact network structure. In contrast, PBS buffer mainly contains monovalent ions, resulting in relatively looser network architectures. In addition, the higher ionic strength and slightly acidic pH of artificial saliva enhance electrostatic shielding and chitosan protonation, thereby strengthening polyelectrolyte interactions and further restricting network expansion [18,20].

Moreover, the swelling ratio decreased progressively with increasing chitosan concentration in both media. The *L. reuteri*-SA/CS1 hydrogel (5 mg·mL^−1^) exhibited the highest swelling capacity, whereas *L. reuteri*-SA/CS5 (15 mg·mL^−1^) showed the lowest. This trend is mainly associated with the increased crosslinking density arising from stronger electrostatic interactions between –NH_3_^+^ (CS) and –COO^−^ (SA), which reduce the network mesh size and limit water diffusion [39]. These findings are consistent with SEM observations (Figure 3A–E), where higher CS content resulted in smaller pore sizes and denser microstructures. The swelling behavior of SA/CS hydrogels is governed by both the ionic environment and the crosslinking density of the polymer network. A higher CS content and ion-rich conditions lead to more compact structures with reduced water uptake, while lower crosslinking densities favor network expansion and increased swelling [40,41]. This tunable swelling property is critical for controlling mass transport and release behavior in hydrogel-based probiotic delivery systems. Since the swelling experiments were conducted under static in vitro conditions, the observed swelling characteristics may not completely reflect the hydrogel performance under the dynamic conditions of the oral cavity.

### 2.5. In Vitro Degradation Behavior

**Figure 7:**
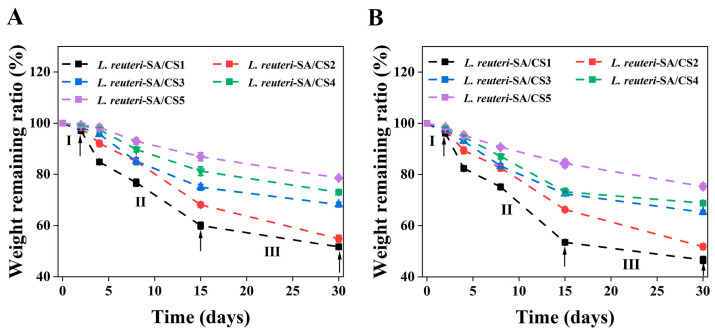
Degradation behavior of *L. reuteri*-SA/CS1-5 hydrogels. (**A**) Degradation in phosphate-buffered saline (PBS) buffer; (**B**) Degradation in artificial saliva. The degradation process was divided into three stages: Stage I (initial hydration, 0–1 d), Stage II (progressive mass loss, 1–15 d), and Stage III (slow degradation, 15–30 d).

The degradation behavior of *L. reuteri*-SA/CS1-5 hydrogels in PBS buffer and artificial saliva is presented in Figure 7A,B. A degradation profile was characterized by an initial hydration stage (stage I, 0–1 d) followed by progressive mass loss (stage II, 1–15 d) and a slower degradation phase at longer incubation times (stage III, 15–30 d). This degradation behavior may be associated with the combined effects of water diffusion, polymer chain relaxation, and gradual crosslink dissociation. Initially, rapid water uptake likely promoted network expansion and solvent penetration into the hydrogel matrix [8]. Subsequently, disruption of ionic crosslinks and weakening of hydrogen bonding interactions may have contributed to partial disruption of the hydrogel network and mass loss [36]. At longer incubation times, the residual network became relatively denser and less accessible to solvent diffusion, resulting in a slower degradation rate.

After 30 d of incubation, the remaining weight ratios of *L. reuteri*-SA/CS1-5 hydrogels ranged from 51.7% to 78.6% in PBS and 46.6% to 75.3% in artificial saliva, indicating favorable long-term structural stability. Although minor differences were observed among formulations with varying CS concentrations, all hydrogels maintained relatively stable degradation profiles. The degradation behavior is also affected by the ionic composition and pH of the surrounding medium. In artificial saliva, multivalent ions may temporarily reinforce the hydrogel network via secondary ionic crosslinking, thereby delaying early-stage degradation [42]. However, the mildly acidic environment promotes chitosan protonation and ion exchange, which accelerates the dissociation of electrostatic interactions over time [37]. In contrast, PBS buffer provides a more stable ionic environment, resulting in a relatively uniform degradation process. Furthermore, increasing CS concentration enhances degradation resistance by increasing crosslinking density and reducing network porosity, thereby limiting water diffusion and bond cleavage. This observation is consistent with previous studies on alginate/chitosan hydrogels, where higher polymer content leads to slower degradation due to a denser and more entangled network structure [18]. Although the hydrogels exhibited favorable structural stability, the degradation experiments were conducted under static in vitro conditions and may not fully represent their behavior in the dynamic oral environment.

### 2.6. Viable Probiotic Availability

**Figure 8:**
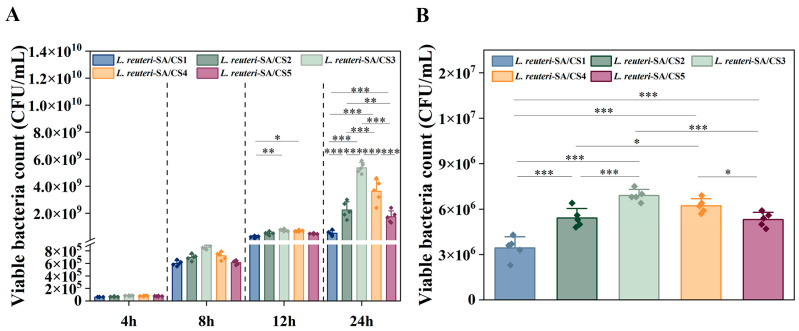
Viable probiotic availability and storage stability of *L. reuteri*-SA/CS1-5 hydrogels. (**A**) Viable probiotic counts during incubation; (**B**) Probiotic viability after 30 days of storage. Statistically significant differences among groups are marked * (*p* < 0.05), ** (*p* < 0.01), or *** (*p* < 0.001).

The viable counts of *L. reuteri* in the culture medium during incubation are shown in Figure 8A. At the initial release stage (4 h and 8 h), no significant differences in viable probiotic counts were observed among the groups (*p* > 0.05). After 12 h, the viable probiotic counts detected in the medium for *L. reuteri*-SA/CS3 and *L. reuteri*-SA/CS4 hydrogels were significantly higher than that from the *L. reuteri*-SA/CS1 group (*p* < 0.05). At 24 h of incubation, the *L. reuteri*-SA/CS3 hydrogel exhibited the highest viable probiotic counts in the medium, with probiotic release significantly higher than that of all other groups (*p* < 0.05). The observed differences in viable probiotic counts may be associated with the internal network structure and mass transfer properties of the hydrogels. These findings suggest that chitosan concentration may regulate probiotic availability by balancing structural protection and mass transport within the hydrogel network. At lower CS concentrations, the relatively loose network facilitates bacterial transport and exchange with the surrounding medium but lacks sufficient structural integrity for sustained release [19]. In contrast, higher CS concentrations lead to increased crosslinking density and reduced mesh size, which limit bacterial migration and mass transfer [43]. The *L. reuteri*-SA/CS3 hydrogel likely achieves an optimal balance between network permeability and structural stability, contributing to higher viable probiotic counts detected in the medium during incubation. It should be noted that the experiment was conducted in MRS medium, which supports bacterial growth. Therefore, the measured CFU values may reflect both probiotic release and subsequent proliferation and should be interpreted as viable probiotic availability rather than precise release kinetics.

After storage at 4 °C in sealed containers for 30 days, all formulations maintained high probiotic viability (Figure 8B). Among them, the *L. reuteri*-SA/CS3 hydrogel showed the highest viable cell count, reaching 6.9 × 10^6^ CFU·mL^−1^, which was significantly higher than that of the other formulations (*p* < 0.05), indicating that the *L. reuteri*-SA/CS3 hydrogel possessed the superior long-term protective effect on probiotic viability. This enhanced viability may be attributed to the protective microenvironment provided by the hydrogel matrix. The SA/CS network can effectively reduce external stress, such as oxygen exposure and moisture loss, while maintaining a hydrated environment favorable for probiotic survival [15]. Moreover, an appropriate crosslinking density, as in the *L. reuteri*-SA/CS3 hydrogel, may facilitate nutrient and metabolite exchange while preventing excessive diffusion of cells, thereby improving long-term probiotic stability. In contrast, overly dense networks may restrict mass transfer, whereas insufficient crosslinking may fail to provide adequate protection [41].

### 2.7. Antibacterial Activity

**Figure 9:**
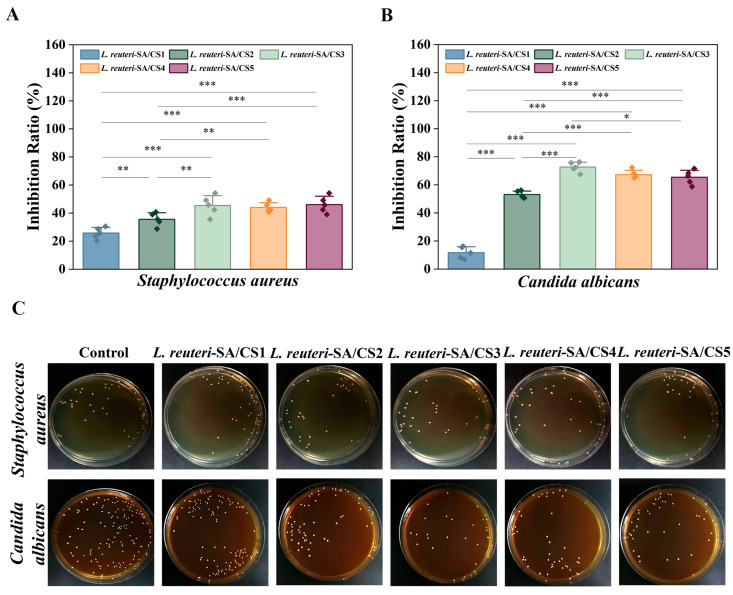
Antibacterial activity of *L. reuteri*-SA/CS1-5 hydrogels. (**A**) Inhibition rates against *Staphylococcus aureus* (*S. aureus*). Statistically significant differences among groups are marked * (*p* < 0.05), ** (*p* < 0.01), or *** (*p* < 0.001); (**B**) Inhibition rates against *Candida albicans* (*C. albicans*); (**C**) Colony formation following co-incubation with *S. aureus* and *C. albicans*.

The inhibitory effects of *L. reuteri*-SA/CS1-5 hydrogels against common oral pathogens were evaluated using the plate count method, and the results are shown in Figure 9A. Compared with the blank control group, all hydrogel formulations effectively reduced the colony counts of *Staphylococcus aureus* (*S. aureus*) and *Candida albicans* (*C. albicans*), indicating their broad-spectrum antibacterial activity. The high concentration groups (*L. reuteri*-SA/CS3-5 hydrogels) showed significantly better inhibitory effects against both pathogens than the low-concentration groups (*L. reuteri*-SA/CS1-2 hydrogels). Specifically, the *L. reuteri*-SA/CS3-5 hydrogels achieved inhibition rates of 35.6–49.1% against *S. aureus* and 65.4–72.7% against *C. albicans* (Figure 9B,C). The antibacterial effect increased with increasing CS concentration up to 10 mg·mL^−1^ (*L. reuteri*-SA/CS3 hydrogel), whereas further increases in CS concentration resulted in only marginal improvements, suggesting a plateau in antibacterial activity. This trend may be attributed to enhanced electrostatic interactions between the positively charged amino groups of CS and negatively charged microbial membranes [39], with the antibacterial effect approaching saturation at higher CS concentrations. Combined with the probiotic availability results, this finding indicates that an intermediate crosslinking density offers a balance between antimicrobial activity, probiotic retention, and mass transport, while a denser network may further enhance probiotic retention and antibacterial efficacy [44].

The antibacterial activity may result from the combined effects of chitosan and sustained *L. reuteri* release. Chitosan can disrupt microbial membranes, while *L. reuteri* may inhibit pathogens through antimicrobial metabolite production, competitive exclusion, and local pH reduction [45,46]. The stronger inhibition against *C. albicans* than *S. aureus* may reflect its greater sensitivity to these factors. As the assays were conducted with planktonic microorganisms, further studies are required to assess antibiofilm activity and clarify the individual roles. Since oral pathogens commonly exist in biofilm-associated communities, the present planktonic results may not fully reflect clinically relevant antibacterial performance, and further studies are required to evaluate antibiofilm activity.

## 3. Conclusions

In this study, *Lactobacillus reuteri*-loaded sodium alginate/chitosan hydrogels were successfully developed via ionic crosslinking as potential bioadhesive platforms for oral mucosal delivery. The hydrogels exhibited well-defined porous architectures, favorable viscoelastic properties, and tunable swelling and degradation behaviors, providing a suitable matrix for probiotic encapsulation. The results demonstrated that chitosan concentration played a critical role in regulating network structure and functional performance. Increasing CS content enhanced crosslinking density, leading to reduced pore size, improved mechanical stability, and restricted swelling capacity, while also influencing degradation kinetics and mass transfer behavior. Notably, the optimized formulation (10 mg·mL^−1^ CS) achieved a balanced network structure that enabled sustained availability of viable probiotics, high post-storage viability, and effective antibacterial activity against *Staphylococcus aureus* and *Candida albicans*. The observed antibacterial performance may be associated with the combined presence of the hydrogel matrix, chitosan, and viable probiotics. However, the individual contribution of each component was not investigated in the present study. Overall, this work establishes a functional hydrogel-based probiotic delivery system suitable for oral mucosal delivery applications, offering a promising microecological strategy for RAU management.

Although the present study demonstrated favorable physicochemical characteristics, sustained availability of viable probiotics, and antibacterial activity, the cytocompatibility, biosafety, and biological effects of the hydrogel on mucosal repair and inflammation were not directly investigated. Future studies will focus on evaluating cell proliferation, migration, anti-inflammatory activity, and therapeutic efficacy in animal models of oral ulcers to further validate its potential for RAU treatment.

## 4. Materials and Methods

### 4.1. Materials and Bacterial Strains

Sodium alginate (SA; viscosity 200 ± 20 mPa·s), chitosan (CS; medium viscosity 200–400 mPa·s; degree of deacetylation > 88%) and acetic acid (purity > 99.5%) were purchased from Macklin (Shanghai, China). Calcium chloride (CaCl_2_; purity > 96%) was obtained from Sinopharm (Shanghai, China). Phosphate-buffered saline (PBS, pH 7.2) was purchased from Servicebio (Wuhan, China). Artificial saliva (pH 6.8) was obtained from Bida (Shenzhen, China).

DeMan, Rogosa and Sharpe (MRS), tryptic soy broth (TSB), and Sabouraud dextrose agar (SDA) media were purchased from Luqiao (Beijing, China). *Lactobacillus reuteri* (*L. reuteri*) PB-LR09 was purchased from Junhe (Xi’an, China). *Staphylococcus aureus* (*S. aureus*) CMCC(B)26003 and *Candida albicans* (*C. albicans*) CMCC(F)98001 were obtained from Luwei (Shanghai, China). The strains were stored at −80 °C in glycerol prior to use.

### 4.2. Preparation of Hydrogel

Chitosan (CS) was dissolved in 1% (*v*/*v*) acetic acid under magnetic stirring to prepare a 1% (*w*/*v*) CS solution, followed by lyophilization to obtain freeze-dried CS powder. Sodium alginate (SA) was dissolved in sterile water under magnetic stirring to prepare a 2% (*w*/*v*) solution. A suspension of *L. reuteri* (10^8^–10^9^ CFU·mL^−1^) was prepared by inoculating the bacteria into MRS medium and incubating at 37 °C for 24 h. After centrifugation of 5 mL of the bacterial suspension (8000 rpm, 30 min), the supernatant was removed, and the resulting pellet washed three times with sterile water. The pellet was resuspended in 2 mL of sterile water and mixed with 2 mL of SA solution. The freeze-dried CS powder at various concentrations (5.0, 7.5, 10.0, 12.5, and 15.0 mg·mL^−1^) was then added to the *L. reuteri*-SA mixture. After gentle stirring to ensure homogeneity, the *L. reuteri*-SA/CS mixture was immersed in a 2% (*w*/*v*) CaCl_2_ solution and cross-linked at 25 °C for 10 min to form five *L. reuteri*-SA/CS hydrogels. The resulting hydrogels were designated as *L. reuteri*-SA/CS1, *L. reuteri*-SA/CS2, *L. reuteri*-SA/CS3, *L. reuteri*-SA/CS4, and *L. reuteri*-SA/CS5, collectively referred to as *L. reuteri*-SA/CS1-5.

### 4.3. Characterization of Hydrogel

The *L. reuteri*-SA/CS1-5 hydrogels were treated with liquid nitrogen, immediately ground into powder, and freeze-dried, sputter-coated with gold, and imaged using a Quanta 250 scanning electron microscope (SEM) (FEI Co., Hillsboro, OR, USA) [47]. The average pore diameter and pore diameter distribution were analyzed from SEM micrographs using ImageJ software (Version 1.54f, National Institutes of Health, USA). Thirty representative pores were randomly selected from each sample, and the pore diameter was measured for quantitative analysis. The mean pore diameter, standard deviation (SD), and coefficient of variation (CV) were calculated based on the measured pore diameters. The chemical structures of *L. reuteri*-SA/CS1-5 hydrogels were characterized by Fourier transform infrared (FTIR) spectroscopy (Thermo Fisher Scientific, Waltham, MA, USA). Spectra were recorded over a wavenumber range of 4000 cm^−1^ to 400 cm^−1^ with a resolution of 4 cm^−1^, accumulating 32 scans [48].

### 4.4. Rheological Property

To evaluate the effect of chitosan concentration on hydrogel rheological behavior, *L. reuteri*-SA/CS1, *L. reuteri*-SA/CS3, and *L. reuteri*-SA/CS5 hydrogels were selected as representative formulations corresponding to low, intermediate, and high chitosan concentrations, respectively. Rheological properties were characterized using a rotational rheometer (MCR 302e, Anton Paar, Graz, Austria) at 37 °C. A strain sweep test (0.01–1000%) was first performed at a fixed angular frequency of 5 rad·s^−1^ to determine the linear viscoelastic region (LVR). Frequency sweep measurements were subsequently conducted at a constant strain of 1% within the LVR over an angular frequency range of 0.01–100 rad·s^−1^. The storage modulus (G′) and loss modulus (G″) were recorded as functions of angular frequency.

### 4.5. Swelling Behavior

The *L. reuteri*-SA/CS1-5 hydrogels (8 mm diameter, 2 mm height) were freeze-dried, and their initial dry weights (*W_i_*) were recorded. The samples were immersed in PBS buffer (pH 7.2) and artificial saliva (pH 6.8) for 24 h at 37 °C with shaking at 90 rpm. Samples were collected at predetermined time intervals (0, 2, 4, 6, 8, 10, 12, and 24 h). The weight of the hydrogels after immersion (*W_f_*) was recorded. The surface moisture of the samples was gently removed using filter paper before weighing. All experiments were conducted in triplicate, and the results are expressed as the mean ± SD. The swelling ratio (*S*) was calculated as follows:

*S* = (*W_f_* − *W_i_*)/*W_i_* × 100% (1)

where *W_i_* and *W_f_* represent the initial and final weights of the hydrogel, respectively.

### 4.6. In Vitro Degradation Behavior

The *L. reuteri*-SA/CS1-5 hydrogels (15 mm diameter, 2 mm height) were lyophilized to a constant weight, and the initial dry weights (*W*_0_) were recorded. The samples were subsequently immersed in 20 mL of PBS buffer (pH 7.2) and artificial saliva (pH 6.8) at 37 °C with shaking at 120 rpm. At predetermined time points (0, 1, 3, 5, 10, 15, 20, and 30 days), the samples were retrieved, gently rinsed with deionized water to remove residual salts, and lyophilized to constant weight (*W_r_*). All experiments were conducted in triplicate, and the results are expressed as the mean ± SD. The weight remaining ratio (*D*) was calculated as follows:

*D* = *W_r_*/*W*_0_ × 100% (2)

where *W*_0_ and *W_r_* represent the initial dry weight and the residual dry weight of the hydrogel after degradation, respectively.

### 4.7. Viable Probiotic Availability

The *L. reuteri*-SA/CS1-5 hydrogels (10 mm diameter, 2 mm height) were inoculated into 10 mL of MRS medium and incubated at 37 °C with shaking at 150 rpm. In total, 20 μL of the co-incubation medium was collected at 4, 8, 12, and 24 h, and spread onto MRS agar plates. The plates were incubated at 37 °C for 48 h, and colony-forming units (CFUs) were counted. All experiments were performed in triplicate, and the results are expressed as the mean ± SD. To evaluate the storage stability, the *L. reuteri*-SA/CS1-5 hydrogels were prepared as described above, sealed, and stored at 4 °C for 30 days. Subsequently, the probiotic release was measured following the same procedure.

### 4.8. Antimicrobial Test

*Staphylococcus aureus* (*S. aureus*) and *Candida albicans* (*C. albicans*) were used as model oral pathogens. *S. aureus* and *C. albicans* were cultured in TSB medium and SDA medium (37 °C, 150 rpm), respectively, until the exponential growth phase, then collected by centrifugation and resuspended in fresh TSB medium (for *S. aureus*) or SDA medium (for *C. albicans*). Subsequently, 5 mL of *S. aureus* (10^8^–10^9^ CFU·mL^−1^) or *C. albicans* suspension (10^5^–10^6^ CFU·mL^−1^) was mixed with the *L. reuteri*-SA/CS1-5 hydrogels (10 mm diameter, 2 mm height). The mixtures were incubated at 37 °C with shaking at 150 rpm for 24 h. After co-incubation, the suspensions were serially diluted, and 100 μL aliquots were spread onto TSB agar (*S. aureus*) or SDA agar (*C. albicans*). The plates were incubated at 37 °C for 24 h (*S. aureus*) or 48 h (*C. albicans*), and CFU were counted. A blank control group without hydrogel was included. All experiments were performed in triplicate, and the results are expressed as the mean ± SD. The inhibition ratio (*I*) was calculated as follows:

*I* = (*C_b_* − *C_t_*)/*C_b_* × 100% (3)

where *C_b_* and *C_t_* represent the viable counts of the blank control and the hydrogel treated groups, respectively.

### 4.9. Statistical Analysis

Data were presented as mean ± standard deviation (SD). Statistical analysis was performed using one-way analysis of variance (ANOVA) followed by the least significant difference (LSD) post hoc test in SPSS 16.0 (SPSS Inc., Chicago, IL, USA) to evaluate differences among groups. A *p*-value < 0.05 was considered statistically significant.

## Data Availability

The data that support the findings of this study are available from the corresponding author upon reasonable request.

## Supplementary Materials

The following supporting information can be downloaded at: https://www.mdpi.com/article/10.3390/gels12070581/s1, Figure S1: Pore diameter distribution histograms of *L. reuteri*-SA/CS1-5 hydrogels obtained by ImageJ analysis of SEM images (*n* = 30). D denotes the mean pore diameter (mean ± SD), and CV denotes the coefficient of variation, calculated as (SD/mean) × 100%.
