# Supplementary material for: Bioadhesive Alginate/Chitosan Hydrogels for Sustained Probiotic Delivery and Enhanced Antibacterial Treatment of Oral Ulcers
Source: Gels. 2026 Jul 1;12(7):581. doi: 10.3390/gels12070581 (PMC13407556; doi:10.3390/gels12070581)
Supplement: Supplementary file 1 [file gels-12-00581-s001.zip › gels-4347925-supplementary.pdf]

Supplementary material

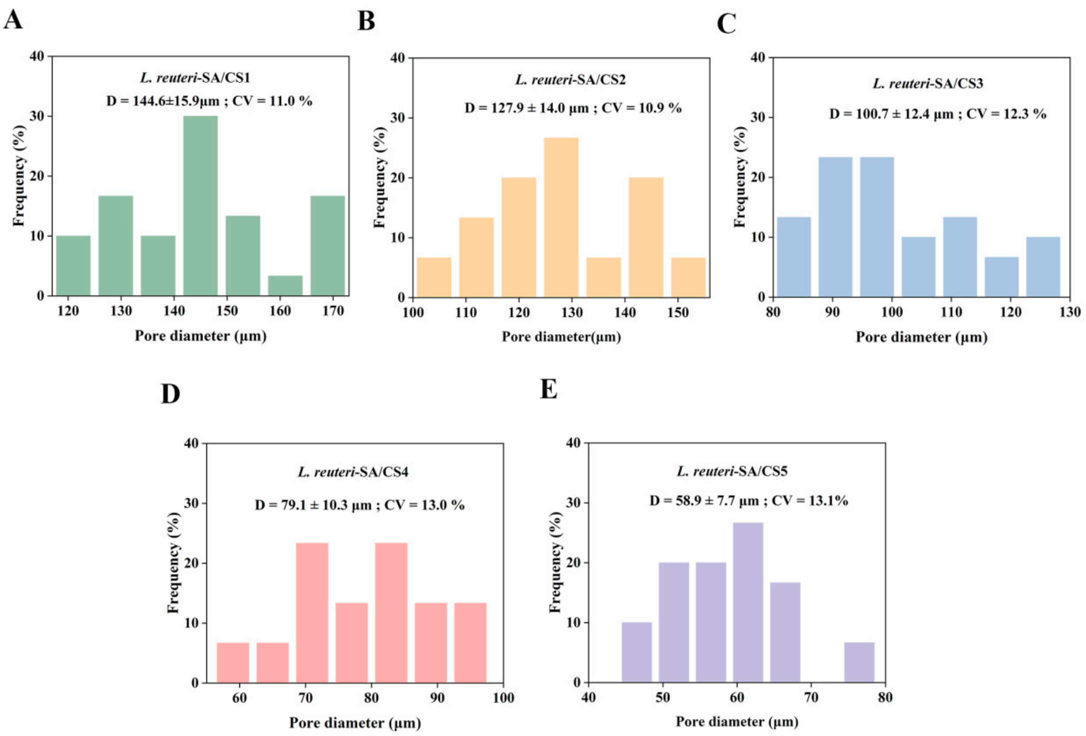

Figure S1. Pore diameter distribution histograms of *L. reuteri*-SA/CS1-5 hydrogels obtained by ImageJ analysis of SEM images (n = 30). D denotes the mean pore diameter (mean ± SD), and CV denotes the coefficient of variation, calculated as (SD/mean) × 100%.
